# Razão Neutrófilo-Linfócito e Aterosclerose da Aorta Abdominal entre Indivíduos Assintomáticos

**DOI:** 10.36660/abc.20201163

**Published:** 2022-01-11

**Authors:** Bárbara Said Marin, Fernando Cesena, Antonio Gabriele Laurinavicius, Raul D. Santos, Marcio Sommer Bittencourt

**Affiliations:** 1 Hospital Israelita Albert Einstein São Paulo SP Brasil Hospital Israelita Albert Einstein,São Paulo, SP – Brasil; 2 Universidade de São Paulo São Paulo SP Brasil Universidade de São Paulo,São Paulo, SP – Brasil

**Keywords:** Aterosclerose, Biomarcadores, Linfócitos, Neutrófilos, Fatores de Risco

## Abstract

**Fundamento:**

A razão neutrófilo-linfócito (RNL) tem sido proposta como um marcador inflamatório possivelmente associado a aterosclerose coronariana, embora a maioria dos dados atuais seja restrita à fase aguda. Além disso, a associação entre a RNL e a aterosclerose extracoronariana ainda não está clara.

**Objetivo:**

Analisar a associação entre a RNL e aterosclerose da aorta abdominal (AtAA).

**Métodos:**

Foram incluídos pacientes assintomáticos submetidos a um programa de rastreamento. A AtAA foi avaliada através de ultrassom. Os números absolutos de leucócitos e linfócitos foram utilizados para calcular a RNL. Foi estabelecido um nível de significância estatística de 0,05.

**Resultados:**

De 36.985 indivíduos (idade: 42±10 anos, 72% homens), foi identificada a presença de AtAA em 7%. Aqueles com AtAA eram mais velhos e tinham maior propensão a serem homens e diabéticos. A presença de AtAA foi associada a RNL aumentada (odds ratio [OR] 1,17; intervalo de confiança de 95% [IC95%] 1,13-1,21). No entanto, a associação deixou de ser significativa quando a análise foi ajustada para os fatores de risco (OR 1,02; IC95% 0,97-1,06), principalmente devido à inclusão da idade no modelo. Quando os neutrófilos e linfócitos foram analisados separadamente, a associação negativa entre os linfócitos e a RNL foi invertida com a inclusão da idade, o que sugere um forte efeito confundidor da idade na relação entre linfócitos e aterosclerose. Por fim, a associação entre os neutrófilos e a AtAA deixou de ser significativa após o ajuste adicional para os fatores de risco tradicionais, mas não apenas para a idade.

**Conclusão:**

Embora a RNL tenha se associado a AtAA, foi principalmente devido ao efeito confundidor da idade. No geral, os resultados sugerem um papel limitado da contagem de leucócitos como biomarcador de AtAA.

## Introdução

As doenças cardiovasculares são a principal causa de morte no mundo.^1^ A combinação de fatores de risco, como diabetes, hipertensão, dislipidemia, obesidade e tabagismo, pode causar o desenvolvimento de aterosclerose. Nos estágios iniciais da formação de placa de ateroma, lipoproteínas de baixa densidade (*low-density lipoproteins*, LDLs) circulantes, no contexto da disfunção endotelial, penetram e se acumulam na túnica íntima das artérias. Quando oxidadas, as partículas de LDL podem dar início a uma resposta inflamatória que culmina no recrutamento de monócitos/macrófagos para a região da placa e ativa a imunidade inata e adaptativa. Portanto, o crescimento e as complicações das placas ateroscleróticas são uma resposta inflamatória imunomediada.^2^

Vários estudos demonstraram a relação entre a contagem de glóbulos brancos e o risco de doença arterial coronariana (DAC).^3^ O estado inflamatório sistêmico leva a um aumento dos neutrófilos, e o estresse agudo provocado por complicações das placas ateroscleróticas leva a uma diminuição dos linfócitos.^4-6^ Os neutrófilos também foram associados a uma chance maior de eventos,^7^ enquanto a taxa de linfócitos foi significativamente menor em pacientes com eventos cardíacos que ainda apresentavam um risco maior de eventos futuros (por exemplo, DAC, angina instável e morte cardíaca).^8,9^ A razão neutrófilo-linfócito (RNL) é um marcador inflamatório que tem sido extensamente estudado nos últimos anos e aparenta ter um papel importante não apenas na predição de eventos cardiovasculares, mas também na predição de desfechos clínicos no contexto de hemorragias cerebrais,^10,11^ eventos cardíacos maiores^12^ e sepse e doenças infecciosas.^13^ Dessa forma, esse simples índice, derivado de um teste de baixo custo e facilmente reproduzível, pode conter informações relevantes sobre o risco de desfechos cardiovasculares.^14^

A associação entre a RNL e prognóstico em diferentes tipos de doença cardiovascular, como em síndromes coronarianas agudas, arritmias cardíacas, insuficiência cardíaca congestiva descompensada, substituição da valva aórtica transcateter e doenças valvulares cardíacas,^15^ tem sido relatada por diversos autores. No entanto, alguns estudos ainda apresentam limitações nas análises multivariadas, que nem sempre consideram todos os fatores confundidores, o que compromete os resultados da verdadeira associação entre a RNL e o risco cardiovascular. Além disso, não há dados disponíveis que correlacionem a RNL com doença cardiovascular aterosclerótica subclínica e apliquem a RNL à estratificação de risco cardiovascular. Assim, os objetivos deste estudo foram correlacionar a presença de aterosclerose subclínica com a RNL e avaliar se a RNL adiciona discriminação aos fatores de risco tradicionais.

## Métodos

### População do estudo

Foram incluídos todos os indivíduos submetidos a um programa de rastreamento no Centro de Medicina Preventiva do Hospital Israelita Albert Einstein, em São Paulo, Brasil, no período de 2006 a 2015. O programa consiste em avaliações clínica e laboratorial extensas e ultrassom abdominal. O protocolo do estudo foi aprovado pelo Conselho de Revisão Institucional, com dispensa de consentimento informado.

### Avaliação clínica e laboratorial

Os dados demográficos, o histórico médico e o uso de medicamentos foram obtidos através de questionários padrões. O estado de tabagismo foi categorizado em fumante (pelo menos um cigarro nos últimos 30 dias) ou ex-fumante e não fumante. A altura (m) e o peso (kg) foram aferidos com um estadiômetro e uma balança médica antropométrica, respectivamente, para calcular o índice de massa corporal (IMC, kg/m^2^). A pressão arterial foi aferida três vezes na posição sentada com um esfigmomanômetro aneroide de acordo com o método padrão recomendado pela *American Heart Association*.^16^ A hipertensão foi definida como níveis de pressão arterial ≥ 140/90 mmHg durante a avaliação ou uso de medicamentos para pressão alta. O diabetes melito foi definido como valores de glicemia de jejum ≥ 5,55 mmol/L ou uso de tratamento medicamentoso para hiperglicemia. A dislipidemia foi definida como nível elevado de triglicerídeos (TG) (≥ 1,7 mmol/L), nível elevado de colesterol LDL (4,12 mmol/L) e baixo nível de colesterol de lipoproteína de alta densidade (*high-density lipoprotein*, HDL) (< 1,02 mmol/L para homens e < 1,28 mmol/L para mulheres) ou uso de medicamentos hipolipemiantes. Foram coletadas amostras de sangue após o jejum recomendado de 12 horas. As amostras foram processadas no laboratório do Centro de Medicina Preventiva do Hospital Israelita Albert Einstein. Os níveis de colesterol total, TG, colesterol HDL, glicose e células sanguíneas foram determinados através de testes laboratoriais automatizados padrões.^17^ O colesterol LDL foi determinado diretamente ou pela fórmula de Friedewald^18^ para níveis de TG ≥ 4,5 mmol/L e < 4,5 mmol/L, respectivamente.

### Ultrassom abdominal

Foi realizado ultrassom abdominal com abordagem padrão por radiologistas certificados. A aorta abdominal foi sistematicamente avaliada para a presença de aterosclerose. Foi definida como aterosclerose da aorta abdominal (AtAA) a presença de placa lipídica ou de ateroma no relatório do ultrassom abdominal.

### Análise estatística

Variáveis contínuas são apresentadas como médias e desvios padrão ou como medianas e intervalos interquartis, conforme apropriado. A normalidade foi avaliada através de inspeção visual de histogramas. As variáveis categóricas são apresentadas como números absolutos e porcentagens. As diferenças nas características basais dos indivíduos de acordo com os quintis da RNL e a presença de AtAA foram avaliadas por meio do teste *t* para amostras independentes; da análise de variância (ANOVA) unidirecional para variáveis contínuas com distribuição normal; e do teste da soma de postos de Wilcoxon ou do teste de Kruskal-Wallis para variáveis com distribuição não normal. O teste do qui-quadrado foi utilizado para as variáveis categóricas. As associações entre RNL, neutrófilos, linfócitos e AtAA foram primeiramente testadas por análise multivariada e posteriormente ajustadas para sexo e idade com modelos de regressão logística. As análises multivariadas adicionais incluíram idade, sexo, tabagismo, hipertensão arterial, diabetes e dislipidemia. Os testes foram conduzidos com um nível de significância de 5%. Todas as análises foram realizadas no *software* Stata, versão 13.0.

## Resultados

A amostra do estudo incluiu 36.985 indivíduos (homens: 71,5%; idade média: 42,3±9,9 anos). As características demográficas, clínicas e laboratoriais basais estão apresentadas na Tabela 1 para todos os pacientes e de acordo com os quintis da RNL.

**Tabela 1:** – Características basais dos participantes do estudo e comparação entre os quintis da razão neutrófilo-linfócito

	Total	Quintil 1	Quintil 2	Quintil 3	Quintil 4	Quintil 5	p
Idade (anos)	42,3±9,9	40,3±9,8	41,6±9,7	42,4±9,7	42,8±9,7	44,4±10,3	< 0,001
Sexo masculino (%)	26.248 (71,5%)	5.144 (70%)	5.430 (74%)	5.365 (73%)	5.317 (72%)	4.992 (68%)	< 0,001
Tabagismo (%)							0,042
Ex-fumante	4.790 (13,1%)	958 (13,1%)	963 (13,1%)	956 (13%)	965 (13,2%)	948 (12,9%)	
Fumante	3.759 (10,3%)	726 (9,9%)	732 (10%)	720 (9,8%)	738 (10,1%)	843 (11,5%)	
Diabetes melito (%)	936 (2,6%)	116 (2%)	157 (2%)	178 (2%)	200 (3%)	285 (4%)	< 0,001
Hipertensão (%)	4.819 (13,1%)	782 (11%)	841 (11%)	963 (13%)	999 (14%)	1.234 (17%)	< 0,001
IMC (kg/m^2^)	26,5±4,3	26,2±4,2	26,4±4,3	26,7±4,3	26,8±4,4	26,5±4,3	0,027
Dislipidemia (%)	9.927 (27%)	1.878 (26%)	1.990 (27%)	2.093 (28%)	1.960 (27%)	2.006 (27%)	0,002
Triglicerídeos* (mg/dL)	112 (79-161)	107 (77-156)	113 (79-163)	112 (81-162)	115 (80-163)	112 (79-158)	< 0,001
Colesterol (mg/dL)	196,9±37,6	198,2±37,7	199±37,3	197,7±38	196,8±37,5	193±37,2	0,328
Colesterol HDL (mg/dL)	49,1±13,6	50,6±14,7	49,1±13,5	48,7±13,3	48,2±13	49±13,6	< 0,001
Colesterol LDL (mg/dL)	121,7±34	121,9±34,3	123,1±33,7	122,8±34,2	122,1±34	118,6±33,4	0,122
Leucócitos (/mm^3^)	6.472±1.575	5.918±1.368	6.202±1.364	6.344±1.397	6.609±1.502	7.286±1.840	< 0,001
Neutrófilos (/mm^3^)	3.600±1.195	2.616±680	3.166±718	3.494±789	3.906±918	4.818±1.399	< 0,001
Linfócitos (/mm^3^)	2.117±580	2.554±615	2.285±515	2.101±473	1.949±452	1.696±430	< 0,001
Proteína C reativa*	0,12 (0,06-0,27)	0,10 (0,05-0,22)	0,11 (0,06-0,23)	0,12 (0,06-0,26)	0,13 (0,07-0,28)	0,17 (0,08-0,38)	< 0,001

*Mediana (intervalo interquartil). IMC: índice de massa corporal; HDL: lipoproteína de alta densidade; LDL: lipoproteína de baixa densidade. Observação: A análise de variância unidirecional (ANOVA) foi utilizada para as variáveis contínuas. O teste de Kruskal-Wallis foi utilizado para os triglicerídeos. O teste do qui-quadrado foi utilizado para as variáveis categóricas.

Não houve diferença entre os quintis da RNL para os níveis de colesterol total e colesterol LDL. Os pacientes no quintil mais alto da RNL eram mais velhos e mais propensos a ter diabetes e hipertensão (p < 0,001 para todos), tendo apresentado a maior contagem de neutrófilos e a menor contagem de linfócitos (p < 0,001 para ambos). Os pacientes no quintil mais baixo da RNL apresentaram o IMC mais baixo (p = 0,027), o nível de TG mais baixo (p < 0,001) e o nível de colesterol HDL mais alto (p < 0,001). Também apresentaram a menor contagem de neutrófilos e a maior contagem de linfócitos (p < 0,001 para ambos).

A AtAA foi identificada por ultrassom em 7% dos pacientes. Em comparação aos participantes sem AtAA, aqueles com AtAA eram mais velhos, mais frequentemente do sexo masculino e fumantes ou ex-fumantes e com maior frequência possuíam um diagnóstico de diabetes, hipertensão ou dislipidemia (Tabela 2).

**Tabela 2:** – Características dos pacientes de acordo com a presença de aterosclerose no ultrassom abdominal

	Aterosclerose	Sem aterosclerose	p
Idade (anos)	57,2±8,3	41,2±9,1	< 0,001
Sexo masculino (%)	2.132 (82%)	24.476 (71%)	< 0,001
Tabagismo (%)			< 0,001
Ex-fumante	810 (31,2%)	4.042 (11,7%)	
Fumante	379 (14,6%)	3.444 (10%)	
Diabetes melito (%)	253 (10%)	700 (2%)	< 0,001
Hipertensão (%)	1.004 (39%)	3.896 (11%)	< 0,001
IMC (kg/m^2^)	27,3±3,8	26,4±4,3	< 0,001
Dislipidemia (%)	1.419 (55%)	8.655 (25%)	< 0,001
Triglicerídeos* (mg/dL)	128 (91-178)	110 (78-159)	< 0,001
Colesterol (mg/dL)	196,3±42	196,9±37,3	0,21
Colesterol HDL (mg/dL)	46,5±12,7	49,2±13,7	< 0,001
Colesterol LDL (mg/dL)	120,7±37,8	121,7±33,7	0,07
Leucócitos (/mm^3^)	6.611,7±1.775,5	6.474,8±1.665,2	< 0,001
Neutrófilos (/mm^3^)	3.732±1.248,6	3.590,7±1.191,1	< 0,001
Linfócitos (/mm^3^)	2.077,8±822,5	2.126,6±592,9	< 0,001

*Mediana (intervalo interquartil). IMC: índice de massa corporal; HDL: lipoproteína de alta densidade; LDL: lipoproteína de baixa densidade; Observação: O teste t foi utilizado para variáveis contínuas. O teste de Mann-Whitney foi utilizado para os triglicerídeos. O teste do qui-quadrado foi utilizado para as variáveis categóricas.

A RNL foi mais alta em pacientes com AtAA em comparação àqueles sem AtAA. Após a análise multivariada, níveis mais elevados de RNL foram diretamente associados a aterosclerose. Quando analisados separadamente, os neutrófilos foram diretamente associados a AtAA, enquanto os linfócitos foram negativamente associados. No entanto, não houve associação entre a RNL e aterosclerose na análise multivariada quando ajustada para sexo, idade e fatores de risco, principalmente devido à inclusão da idade. A associação negativa entre linfócitos e AtAA foi revertida com a inclusão da idade no modelo, o que sugere a presença de um efeito confundidor. A associação entre neutrófilos e AtAA deixou de ser significativa após o ajuste para os fatores de risco tradicionais, mas não apenas para a idade.

## Discussão

O presente estudo demonstrou que não há associação entre RNL e aterosclerose aórtica após a consideração dos confundidores conhecidos. Apesar da associação significativa observada na análise univariada, esses efeitos parecem estar amplamente relacionados ao efeito confundidor da idade, uma vez que a RNL está fortemente correlacionada à idade nesta população. Coletivamente, este estudo sugere que a RNL não serve como um marcador de aterosclerose em pacientes assintomáticos que participaram de um programa de rastreamento.

**Figura 1:**
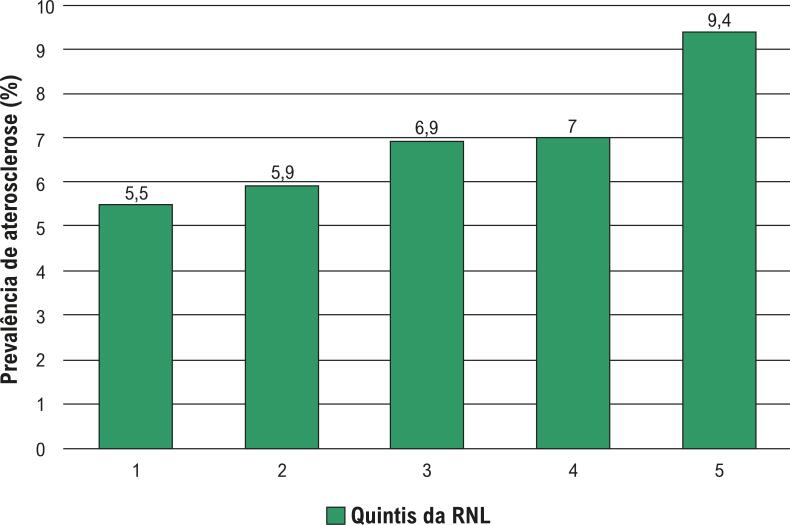
– Prevalência de aterosclerose de acordo com os quintis da razão neutrófilo-linfócito (RNL) (p < 0,001).

Já se sabe que os biomarcadores de inflamação estão associados a um risco maior de eventos cardiovasculares e que algumas terapias anti-inflamatórias são capazes de preveni-los.^19^ A identificação de pacientes de alto riso é fundamental para explorar o tratamento ideal, sendo que a RNL pode ser um importante biomarcador a ser identificado nesses pacientes, a qual está associada ao prognóstico em doenças ateroscleróticas, bem como em sua prevalência, como aponta a Figura 1.

A associação entre a RNL como um preditor de mortalidade e desfechos coronarianos agudos foi demonstrada por vários estudos. Em doenças agudas, os resultados estão associados a níveis elevados de neutrófilos,^12^ os mediadores de respostas a lesões miocárdicas como infarto do miocárdio. Esse resultado também foi demonstrado em estudos sobre doença coronariana estável. A contagem de linfócitos relativa está associada à sobrevida de pacientes com DAC,^9^ enquanto outros biomarcadores, como proteína C reativa (PCR) e leucócitos, estão associados a desfechos crônicos e agudos.^20^ A PCR, como a RNL, é um biomarcador associado a inflamação e predição de risco de mortalidade. Em modelos de estudo com apenas RNL ou PCR, cada parâmetro isolado foi capaz de predizer o risco. No entanto, quando ambos os biomarcadores foram aplicados, houve uma melhora significativa na predição.^21^ Em contrapartida, os resultados deste estudo não corroboram esses achados. Embora os resultados de outros estudos afirmem que a RNL seja um preditor independente de mortalidade cardiovascular, a presente análise demonstrou que há um forte fator confundidor relacionado à inclusão da idade no modelo.

Como a RNL se correlaciona à idade do paciente, a análise deve ser ajustada de acordo. No entanto, até o momento, não encontramos nenhum estudo que tenha realizado tal ajuste. Todas as análises são baseadas em fatores de risco e prognóstico. Como a idade é um ponto importante de comparação entre os pacientes, o ajuste é extremamente necessário.

Há diferenças entre a população do presente estudo e de outros estudos. Foi analisado um grupo mais jovem de uma grande população, o qual possuía boas condições socioeconômicas e consistia principalmente em homens e pessoas brancas. A maioria dos estudos incluem populações do hemisfério Norte, enquanto o presente estudo incluiu uma população de um país tropical da América Latina. Além disso, foram realizados testes laboratoriais e avaliação sistemática dos fatores de risco. Na análise estatística, foram realizados ajustes detalhados para os fatores confundidores. A RNL, os neutrófilos e os linfócitos foram analisados separadamente (Tabela 3).

**Tabela 3:** – Análise multivariada da relação entre a razão neutrófilo-linfócito, neutrófilos ou linfócitos e aterosclerose abdominal

*Odds ratio* para aterosclerose (intervalo de confiança de 95%)			
	Não ajustado	Modelo 1	Modelo 2
RNL	1,17 (1,13-1,21)	1,00 (0,96-1,05)	1,00 (0,95-1,04)
Neutrófilo	1,07 (1,03-1,11)	1,05 (1,01-1,10)	0,99 (0,95-1,04)
Linfócito	0,91 (0,87-0,95)	1,06 (1,02-1,11)	1,01 (0,97-1,05)

*RNL: razão neutrófilo-linfócito. Modelo 1: Ajustado para idade e sexo. Modelo 2: Ajustado para idade, sexo, tabagismo, hipertensão, diabetes e dislipidemia.*

O presente estudo deve, no entanto, ser compreendido no contexto do seu desenho. Devido ao caráter transversal dos dados, não foi possível inferir causalidade. A população selecionada incluiu uma prevalência maior de homens, em sua maioria jovens, o que leva a uma baixa prevalência de doença e pode atenuar a capacidade de identificar associações. Além disso, o foco do presente estudo foi a avaliação de aterosclerose aórtica, que não necessariamente possui o mesmo processo fisiopatológico da aterosclerose em outros locais, como a artéria coronária.

## Conclusão

Embora tenha havido associação entre aterosclerose e RNL, foi principalmente devido ao efeito confundidor da idade. A associação entre neutrófilos e linfócitos e aterosclerose deixou de ser significativa ao serem incluídos em modelos multivariados. Os resultados sugerem um papel limitado do biomarcador na avaliação de aterosclerose subclínica.
