# Supplementary material for: Razão Neutrófilo-Linfócito e Aterosclerose da Aorta Abdominal entre Indivíduos Assintomáticos
Source: Arq Bras Cardiol. 2022 Jan 11;118(4):729–34. [Article in Portuguese] doi: 10.36660/abc.20201163 (PMC9007006; doi:10.36660/abc.20201163)
Supplement: Translation

# Translation: Neutrophil-To-Lymphocyte Ratio and Abdominal Aortic Atherosclerosis among Asymptomatic Individuals

**Authors:** Bárbara Said Marin, Fernando Cesena, Antonio Gabriele Laurinavicius, Raul D. Santos, Marcio Sommer Bittencourt

**Affiliations:** 1 Brazil Hospital Israelita Albert Einstein, São Paulo, SP – Brazil; 2 Brazil Universidade de São Paulo, São Paulo, SP – Brazil

**Keywords:** Atherosclerosis, Biomarkers, Lymphocytes, Neutrophils, Risk Factors

## Abstract

**Background:**

Neutrophil-to-lymphocyte ratio (NLR) has been proposed as an inflammatory marker that might be associated with coronary atherosclerosis, although most of the current data is restricted to the acute setting. Additionally, the association of NLR with extracoronary atherosclerosis and stable disease remains unclear.

**Objective:**

To analyze the association between NLR and abdominal aortic atherosclerosis (AAAt).

**Methods:**

We included asymptomatic individuals who underwent a health screening program. AAAt was measured by ultrasound. Absolute leukocyte and lymphocyte counts were used to calculate the NLR. The level of significance for statistical analysis was 0.05.

**Results:**

Among 36,985 individuals (age: 42±10 years, 72% male), AAAt was identified in 7%. Those with AAAt were older and more likely to be male and diabetic. Presence of AAAt was associated with increased NLR (odds ratio [OR] 1.17; 95% confidence interval [CI] 1.13-1.21). However, this association was no longer significant when the analysis was adjusted for risk factors (OR 1.02; 95% CI 0.97-1.06), mostly due to the inclusion of age in the model. When neutrophils and lymphocytes were analyzed separately, the negative association between lymphocytes and AAAt was inverted once age was accounted for, suggesting a strong confounding effect of age on the relationship between lymphocytes and atherosclerosis. Finally, the association of neutrophils and AAAt lost significance after an additional adjustment for traditional risk factors, but not age alone.

**Conclusion:**

Although the NLR was associated with AAAt, this was largely due to the confounding effect of age. Overall, the results suggest a limited role of leukocyte measurements as biomarkers of AAAt.

## Introduction

Cardiovascular diseases (CVDs) are the leading cause of death worldwide.^1^ Combinations of risk factors, such as diabetes, hypertension, dyslipidemia, obesity, and smoking, can lead to the development of atherosclerosis. In the early stages of atheroma plaque formation, circulating low-density lipoproteins (LDLs), in the context of endothelial dysfunction, penetrate and accumulate in the tunica intima of the arteries. When oxidized, LDL particles may initiate an inflammatory response that culminates with the recruitment of monocytes/macrophages to the plaque region and activate innate and adaptive immunity. Therefore, the growth and complications of atherosclerotic plaques are an immune-mediated inflammatory response.^2^

Many studies have noted the relationship between white blood cell (WBC) count and coronary artery disease (CAD) risk.^3^ The systemic inflammatory state leads to an increase in neutrophils, and the acute stress caused by complications of atherosclerotic plaques leads to a decrease in lymphocytes.^4-6^ Neutrophils were also associated with a higher chance of events,^7^ while lymphocytes were significantly lower in patients with cardiac events and who still had a higher risk of future events (eg, CAD, unstable angina, cardiac death).^8,9^ The neutrophil-to-lymphocyte ratio (NLR) is an inflammatory marker that has been extensively studied in recent years and appears to play an important role not only in predicting cardiovascular events but also in predicting clinical outcomes in the setting of cerebral hemorrhages,^10,11^ major cardiac events,^12^ and sepsis and infectious diseases.^13^ Therefore, this simple index, derived from an inexpensive and easily reproducible test, may contain significant information regarding the risk of cardiovascular outcomes.^14^

The association between NLR and prognosis in different settings of cardiovascular disease, such as acute coronary syndromes, cardiac arrhythmias, congestive heart failure decompensation, transcatheter aortic valve replacement, and valvular heart diseases,^15^ has been reported by several authors. However, some studies still have limitations in multivariate analyses that do not always consider all the confounding factors, impairing the results of the true association between NLR and cardiovascular risk. Moreover, there are no data available correlating NLR with subclinical atherosclerotic cardiovascular disease and showing the application of NLR in cardiovascular risk stratification. Thus, the objectives of this study were to correlate the presence of subclinical atherosclerosis with NLR and to assess whether NLR adds discrimination to traditional risk factors.

## Methods

### Study population

We included all individuals who underwent a health screening program at the Preventive Medicine Center of Hospital Israelita Albert Einstein in São Paulo, Brazil, between 2006 and 2015. This program consists of an extensive clinical and laboratory evaluation and abdominal ultrasonography. The study protocol was approved by the local Institutional Review Board and was granted a waiver of informed consent.

### Clinical and laboratory assessment

Demographics, medical history, and medication use were routinely recorded by standardized questionnaires. Smoking status was categorized as current smoker (at least 1 cigarette during the last 30 days) versus former smoker and nonsmoker. Height (m) and weight (kg) were measured with a stadiometer and a standard physician’s scale, respectively, to calculate body mass index (BMI, kg/m^2^). Blood pressure was measured three times in sitting position with an aneroid sphygmomanometer according to the standard method recommended by the American Heart Association.^16^ Hypertension was defined as average blood pressure values ≥ 140/90 mm Hg during the evaluation or use of blood pressure-lowering medications. Diabetes mellitus was defined as fasting blood glucose level ≥ 5.55 mmol/L or use of drug treatment for hyperglycemia. Dyslipidemia was defined as elevated triglyceride (TG) level (≥ 1.7 mmol/L); elevated LDL-cholesterol level (4.12 mmol/L); and low high-density lipoprotein (HDL)-cholesterol level (< 1.02 mmol/L for men or < 1.28 mmol/L for women) or use of lipid-lowering medications. Blood samples were collected after recommended 12-h fasting and processed at the Central Laboratory of the Preventive Medicine Unit of Hospital Israelita Albert Einstein. Total cholesterol, TG, HDL-cholesterol, glucose, and blood cells were determined with standardized automated laboratory tests.^17^ When TG < 4.5 mmol/L, LDL-cholesterol was calculated by the Friedewald formula.^18^ When TG ≥ 4.5 mmol/L, LDL-cholesterol was measured directly.

### Abdominal ultrasonography

Abdominal ultrasonography was performed by certified radiologists using a standardized approach. The abdominal aorta was systematically evaluated for the presence of atherosclerosis. Abdominal aortic atherosclerosis (AAAt) was defined by the presence of atheroma or lipid plaque in the abdominal ultrasound report.

### Statistical analysis

Continuous variables are presented as means and standard deviations or medians and interquartile ranges, as appropriate. Normality was assessed by visual inspection of histograms. Categorical variables are presented as absolute counts and percentages. Differences in baseline characteristics of individuals according to NLR quintiles and the presence of AAAt were evaluated with independent-sample t-test, one-way analysis of variance (ANOVA) for continuous variables with a normal distribution, and Wilcoxon rank-sum or Kruskal Wallis test for those known not to be normally distributed. Chi-square test was used for categorical variables. The associations between NLR, neutrophils, lymphocytes, and AAAt were tested first in a univariate analysis and then adjusted for age and gender with logistic regression models. Additional multivariate analyses included age, gender, smoking status, hypertension, diabetes, and dyslipidemia. Tests were conducted at a significance level of 5%. All analyses were performed with Stata version 13.0.

## Results

The study sample consisted of 36,985 individuals (men: 71.5%; mean age: 42.3±9.9 years). Their baseline demographic, clinical, and laboratory characteristics are presented in Table 1, for all the patients and according to NLR quintiles.

**Table 1:** – Baseline characteristics of study participants and comparison between neutrophil-to-lymphocyte ratio quintiles

	Total	Quintile 1	Quintile 2	Quintile 3	Quintile 4	Quintile 5	p
Age (years)	42.3±9.9	40.3±9.8	41.6±9.7	42.4±9.7	42.8±9.7	44.4±10.3	< 0.001
Male (%)	26248 (71.5%)	5144 (70%)	5430 (74%)	5365 (73%)	5317 (72%)	4992 (68%)	< 0.001
Smoking (%)							0.042
Former	4,790 (13.1%)	958 (13.1%)	963 (13.1%)	956 (13%)	965 (13.2%)	948 (12.9%)	
Current	3759 (10.3%)	726 (9.9%)	732 (10%)	720 (9.8%)	738 (10.1%)	843 (11.5%)	
Diabetes mellitus (%)	936 (2.6%)	116 (2%)	157 (2%)	178 (2%)	200 (3%)	285 (4%)	< 0.001
Hypertension (%)	4819 (13.1%)	782 (11%)	841 (11%)	963 (13%)	999 (14%)	1234 (17%)	< 0.001
BMI (kg/m^2^)	26.5±4.3	26.2±4.2	26.4±4.3	26.7±4.3	26.8±4.4	26.5±4.3	0.027
Dyslipidemia (%)	9927 (27%)	1878 (26%)	1990 (27%)	2093 (28%)	1960 (27%)	2006 (27%)	0.002
Triglycerides* (mg/dL)	112 (79-161)	107 (77-156)	113 (79-163)	112 (81-162)	115 (80-163)	112 (79-158)	< 0.001
Cholesterol (mg/dL)	196.9±37.6	198.2±37.7	199±37.3	197.7±38	196.8±37.5	193±37.2	0.328
HDL-cholesterol (mg/dL)	49.1±13.6	50.6±14.7	49.1±13.5	48.7±13.3	48.2±13	49±13.6	< 0.001
LDL-cholesterol (mg/dL)	121.7±34	121.9±34.3	123.1±33.7	122.8±34.2	122.1±34	118.6±33.4	0.122
Leukocytes (/mm^3^)	6472±1,575	5918±1368	6202±1364	6344±1397	6609±1502	7286±1840	< 0.001
Neutrophils (/mm^3^)	3600±1195	2616±680	3166±718	3494±789	3906±918	4818±1399	< 0.001
Lymphocytes (/mm^3^)	2117±580	2554±615	2285±515	2101±473	1949±452	1696±430	< 0.001
C-reactive protein*	0.12 (0.06-0.27)	0.10 (0.05-0.22)	0.11 (0.06-0.23)	0.12 (0.06-0.26)	0.13 (0.07-0.28)	0.17 (0.08-0.38)	< 0.001

*Median (interquartile range). BMI: body mass index; HDL: high-density lipoprotein; LDL: low-density lipoprotein. Note: One-way analysis of variance (ANOVA) was used for continuous variables. Kruskal-Wallis test was used for triglycerides. Chi-square test was used for categorical variables.

There was no difference across NLR quintiles for levels of total cholesterol and LDL-cholesterol. Patients in the highest NRL quintile were older and more likely to have diabetes and hypertension (p < 0.001 for all). They had both the highest neutrophil and lowest lymphocyte counts (p < 0.001 for both). Patients in the lowest NRL quintile had the lowest BMI (p = 0.027), the lowest TG level (p < 0.001), and the highest HDL-cholesterol level (p < 0.001). This group also had the lowest neutrophil and highest lymphocyte counts (p < 0.001 for both).

AAAt was identified by ultrasound in 7% of the patients. Compared with participants without AAAt, those with AAAt were older, more frequently male and former or current smokers, and more often had a diagnosis of diabetes, hypertension, or dyslipidemia (Table 2).

**Table 2:** – Characteristics of patients according to the presence of atherosclerosis on abdominal ultrasonography

	Atherosclerosis	No atherosclerosis	p
Age (years)	57.2±8.3	41.2±9.1	< 0.001
Male (%)	2132 (82%)	24476 (71%)	< 0.001
Smoking (%)			< 0.001
Former	810 (31.2%)	4042 (11.7%)	
Current	379 (14.6%)	3444 (10%)	
Diabetes mellitus (%)	253 (10%)	700 (2%)	< 0.001
Hypertension (%)	1004 (39%)	3896 (11%)	< 0.001
BMI (kg/m^2^)	27.3±3.8	26.4±4.3	< 0.001
Dyslipidemia (%)	1419 (55%)	8655 (25%)	< 0.001
Triglycerides* (mg/dL)	128 (91-178)	110 (78-159)	< 0.001
Cholesterol (mg/dL)	196.3±42	196.9±37.3	0.21
HDL-cholesterol (mg/dL)	46.5±12.7	49.2±13.7	< 0.001
LDL-cholesterol (mg/dL)	120.7±37.8	121.7±33.7	0.07
Leukocytes (/mm^3^)	6611.7±1775.5	6474.8±1665.2	< 0.001
Neutrophils (/mm^3^)	3732±1248.6	3590.7±1191.1	< 0.001
Lymphocytes (/mm^3^)	2077.8±822.5	2126.6±592.9	< 0.001

*Median (interquartile range). BMI: body mass index; HDL: high-density lipoprotein; LDL: low-density lipoprotein. Note: T-test was used for continuous variables. Mann-Whitney test was used for triglycerides. Chi-square test was used for categorical variables.

The NLR was higher in patients with AAAt compared with those without AAAt. After multivariate analysis, higher NLR levels were directly associated with atherosclerosis. When analyzed separately, neutrophils were directly associated with AAAt, whereas lymphocytes were negatively associated with it. However, the association between NLR and atherosclerosis was lost when adjusted for sex, age, and risk factors. This occurred mainly because of the inclusion of age in multivariate analysis. The negative association between lymphocytes and AAAt was reversed when age was included in the model, suggesting a confounding effect. The association between neutrophils and AAAt lost significance after adjustment for traditional risk factors, but not age alone.

## Discussion

This study demonstrated that no association between NLR and aortic atherosclerosis is noted once known confounders are accounted for. Despite the significant association noted in univariate analysis, these effects seemed to be largely related to the confounding effect of age, as NLR strongly correlated with age in our population. Collectively, our study suggests there is no role for NLR as a marker of atherosclerosis in asymptomatic patients who participated in a health screening program.

We already know that inflammation biomarkers are associated with an increased risk of cardiovascular events and some anti-inflammatory therapies are able to prevent them.^19^ Identifying the patients who are at highest risk is key for the best therapy to be explored, and an important biomarker to be identified in these patients could be NLR, which is associated with prognosis in atherosclerotic diseases, as well as its prevalence, as shown in Figure 1.

**Figure 1:** – *Prevalence of atherosclerosis according to neutrophil-to-lymphocyte ratio (NLR) quintiles (p < 0.001).*

The association between NLR as a predictor of mortality and acute coronary outcomes has been demonstrated by many studies. In acute diseases, the results are associated with elevated levels of neutrophils,^12^ the mediators of myocardial injury responses such as myocardial infarction. This has also been demonstrated in studies addressing stable coronary disease. The relative lymphocyte count is associated with the survival of patients with CAD,^9^ while different biomarkers, such as C-reactive protein (CRP) and leukocytes, are associated with chronic and acute outcomes.^20^ CRP, like NLR, is a biomarker associated with inflammation and prediction of mortality risk. In study models including only NLR or CRP, each parameter alone was able to predict risk. When both were applied, however, there was a significant improvement in prediction.^21^ Our results, however, do not support that. While results from other studies claim that NLR is an independent predictor of cardiovascular mortality, our analyses showed that there is a strong confounding factor when age is included in the model.

Since NLR correlates with the patient’s age, the study analysis should be adjusted for it. In no study to date, however, we have observed such an adjustment. All analyses are based on risk factors and prognosis. Because age is an important point of comparison between patients, adjustment is extremely necessary.

There are differences between our study population and those of other studies. We approached a younger group from a large population, and this group had good socioeconomic conditions and consisted mostly of men and White people. Most studies address populations from the Northern Hemisphere, while our population lives in a tropical country in Latin America. Also, our study included the systematic evaluation of risk factors and laboratory tests. In statistical analysis, we made detailed adjustments for confounding factors and performed separate analyses for NLR, neutrophils, and lymphocytes (Table 3).

**Table 3:** – Multivariate analysis of the relationship between neutrophil-to-lymphocyte ratio, neutrophils, or lymphocytes and abdominal atherosclerosis

Odds ratio for atherosclerosis (95% confidence interval)			
	Unadjusted	Model 1	Model 2
NLR	1.17 (1.13-1.21)	1.00 (0.96-1.05)	1.00 (0.95-1.04)
Neutrophil	1.07 (1.03-1.11)	1.05 (1.01-1.10)	0.99 (0.95-1.04)
Lymphocyte	0.91 (0.87-0.95)	1.06 (1.02-1.11)	1.01 (0.97-1.05)

*NLR: neutrophil-to-lymphocyte ratio. Model 1: Adjusted for age and gender. Model 2: Adjusted for age, gender, smoking status, hypertension, diabetes, and dyslipidemia.*

Our study must, however, be read within the context of its design. Our data are cross-sectional, so we were unable to infer causality. The selected population showed a higher prevalence of men, mostly young, which leads to a low prevalence of the disease and may attenuate the ability to perceive associations. In addition, our study focused on the evaluation of aortic atherosclerosis, which does not necessarily have the same pathophysiological process of atherosclerosis in other territories, such as the coronary artery.

## Conclusion

Although atherosclerosis was associated with NLR, this was largely due to the confounding effect of age. The association of neutrophils and lymphocytes with atherosclerosis lost significance once these were included in multivariate models. The results suggest a limited role of the biomarker in the evaluation of subclinical atherosclerosis.
